# Experimental and numerical investigation of a cold-formed steel system used to restore old buildings floor

**DOI:** 10.1038/s41598-024-81674-7

**Published:** 2024-11-30

**Authors:** Ahmed Shamel Fahmy, Sherine Mostafa Swelem, Rowida Saad Farrag, Wael F. M. Mobarak

**Affiliations:** 1 Department of Civil Engineering, Faculty of Engineering, Alexandria University, Alexandria, Egypt; 2 Department of Constructing Engineering, Higher Institute of Engineering and Technology, Alexandria, Egypt; 3 Department of Civil Engineering, University of Business & Technology, Jeddah, Saudi Arabia

**Keywords:** Restoration, Cold formed, Flooring system, Experimental, Steel decking, Civil engineering, Mechanical engineering, Engineering

## Abstract

This paper presents a novel configuration of built-up cold-formed steel (CFS) flooring system in the shape of a box section. A new technique is applied to produce the components of the flooring system, which are fastened by self-drilling screws. This box section consists of a cast-in-situ concrete slab, trapezoidal steel decking, two sigma section, steel plate and stiffening equal angles. The main objectives of this system is to enable rapid construction and decrease the time, requirements, and cost. As a result, the proposed system is designed to use the decking in a longitudinal direction. Many old buildings have sturdy structures but their floors were ruined due to being fabricated from timber. This flooring system will be implemented to increase their quality of life and be reused. The loading experiments of four specimens were carried out. The failure modes of the CFS flooring system, load-deflection relation curves, longitudinal strain distribution at different heights were obtained. The experimental results show that the flooring system has high stiffness and flexural performance and can reach ultimate strength without local buckling failure. The failure occurs due to distortion at the end supports. Then, the capacity of the flooring system was calculated theoretically. Then, the practical and theoretical results were compared. The calculated results agree well with the test results. A three-dimensional finite element model is also established to investigate structural performance of the proposed system.

## Introduction

Building restoration are becoming an increasingly important economic and social demand and a growing part of the global construction industry in all countries. There are many reasons for this, but essentially it is about the fundamental need to preserve the heritage of cities. There is a lot of research focused on designing new solutions and developing new methods for restoration buildings^1^. All materials are compared with standard structural steel in the restoration work, the latter being the most frequently chosen material^2^. Recently, the demand for higher capacity CFS sections has increased in the renovation of buildings^3–5^. The CFS sections did not require the installation and removal of formwork and shoring, which account for part of the cost and require a lot of equipment^6,7^. The usage of cold-formed steel in different schemes, including composite cold-formed steel, built-up sections, and concrete structures, has been investigated by several researchers^8–12^. Furthermore, the new research in applying CFS members leads to connecting several single sections with steel decking to form “built-up” cross-Sect^13^. It is commonly used due to the advantages of high load-carrying capacity and ease of connection. The usage of cold-formed steel sigma section as a beam member has recently gained interest. Its cross-section has a higher web stiffness, which improves its performance in resisting loads. The lip acts as an edge stiffener for the slender flange elements. Stiffeners are usually formed in sections to provide post-buckling strength by stress redistribution and extra strength under compression. Furthermore, the sigma section is light and economical^14–18^. Also, the cold-formed steel decking profiles have gained popularity in steel-concrete composite slabs as they can offer significant savings in cost and construction time by not relying on formwork during construction^19,20^. Steel decking has recently become a popular choice for lightweight structural systems due to the considerable out-of-plane stiffness resulting from corrugations^21^. To develop the required effect between concrete and steel deck, the steel must be able to resist the longitudinal slip and the vertical separation between concrete and steel deck. Only the adhesion between the steel sheet and the concrete is sufficient to create the correct bonding effect in the deck. An effective bond can be achieved by mechanical interlocking through the embossments. The position of the embossing generally depends on the available pressing areas and the quality of the steel sheet material Several studies have investigated steel decking for steel-concrete composite slabs and focused on using the conventional deck in the transverse direction. Although in some cases, due to site constraints and circumstances, it is necessary to install steel decking in a longitudinal direction. Some studies that were rarely considering longitudinal cases have shown that the transverse direction corresponding to the longitudinal direction has a similar flexural performance^22^. Their results depended on that the slab element had some weakness because the steel decking’s ribs were placed transversely (parallel to the direction of the applied load). It is important to investigate over and study the longitudinal direction case to obtain accurate results and provide enough data for those who will use it based on their site condition, as there aren’t many studies that address these longitudinal cases for built-up systems. The built-up sections are generally joined using fasteners such as screws, bolts, welds, and clinches^23^. Self-drilling screws (SDS) connect these elements to create light-weight and easily built structural systems. SDS could be a feasible alternative for other economic and effective connections. They are widely used and require no prefabrication procedures or skilled supervision as they drill the required hole^24^. Using cold-formed sections as built-up beams in floor systems provides high flexibility in design and several advantages^25^. It can to decrease the slab depth, freedom in the design of cross-sections, easy adaptation to irregular geometries and ready availability of sections and materials. Moreover, it is advantageous due to its low price and effort^26^. In addition, cold-formed steel sections are an attractive option for the construction of flooring systems in commercial and residential buildings due to their ease of prefabrication, rapid on-site assembly and high strength-to-weight ratio. Also, the combination of CFS with composite slabs can provide an efficient floor system. Cold-formed, built-up structural systems are increasingly used in the construction industry and are becoming the subject of intensive research by the world’s leading universities and companies because of their efficient material usage^27,28^. In addition, many companies aim to manufacture new alternative floor systems for the construction market^29,30^.

**Fig. 1:**
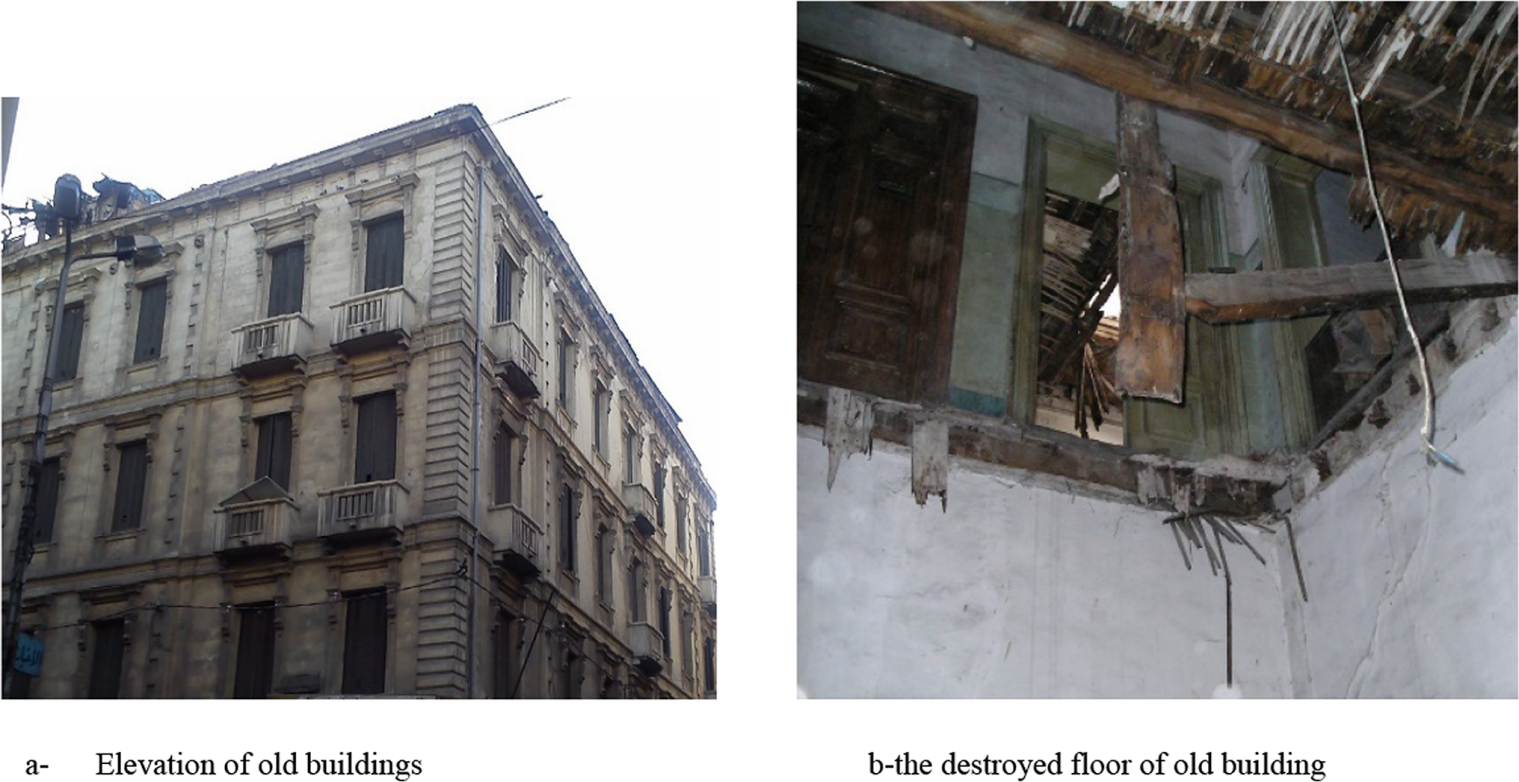
The ruined old building.

Many old buildings have sturdy structures, but their floors were originally built of timber, and have been destroyed over time. As a result, it may be necessary to reuse the old building and extend its life, thus the floors must be restored to improve their endurance^31^.This study was carried out on one of the old buildings, which was built around 100 years ago as shown in Fig. 1a. This study’s main objective is to restore old buildings’ floors as illustrated in Fig. 1b.

There are various problems with restoring this building and many other old buildings. These flooring were composed of timber and have been ruined. It must be restored, but timber is now unavailable and difficult to transport and install. Thus, it was preferable to use built-up steel components that could be assembled in factories. Also, this building is located on an overcrowded main roadway; therefore, cranes are not permitted in this region. Consequently, there is difficulty transporting and storing equipment. Therefore, there will be reliance on manpower to lift and store steel sections. Moreover, there is little space for the use of equipment, and mobility on the damaged roofs is restricted.

Subsequently, it was supposed to propose an alternative idea for solving all the problems and restoring the floor to reuse this building. This idea must be studied to achieve reduced formwork and shoring, lightweight and easy handle, quick assembly, less equipment, design flexibility, and durability.

**Fig. 2:**
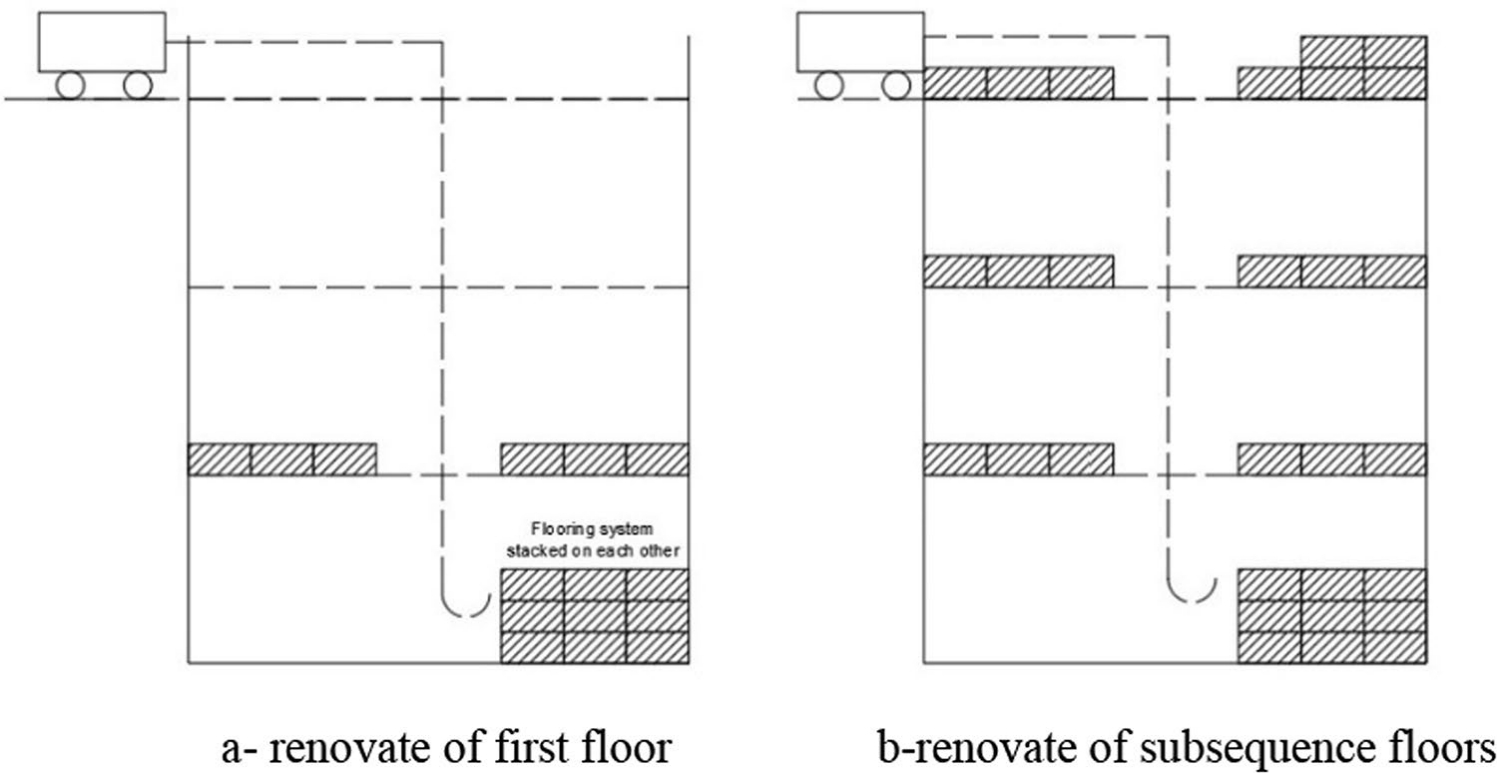
Sketch of floor renovation.

Therefore, the new flooring must consist of many units assembled in the factory and transported to the construction site. Thus, they are transported once to the site and stacked on each other. Figure 2 depicts a sketch of how to renovate the destroyed floor. The manual crane will be installed on the roof because the floor was not destroyed. Each unit will be lifted by a manual crane. They will be arranged side by side as illustrated in Fig. 2a. The assembled units will be put on the first floor, leaving enough space for the crane to lift the other units to subsequent floors as shown in Fig. 2b. After the floor is finished, the concrete will be poured.

**Fig. 3:**
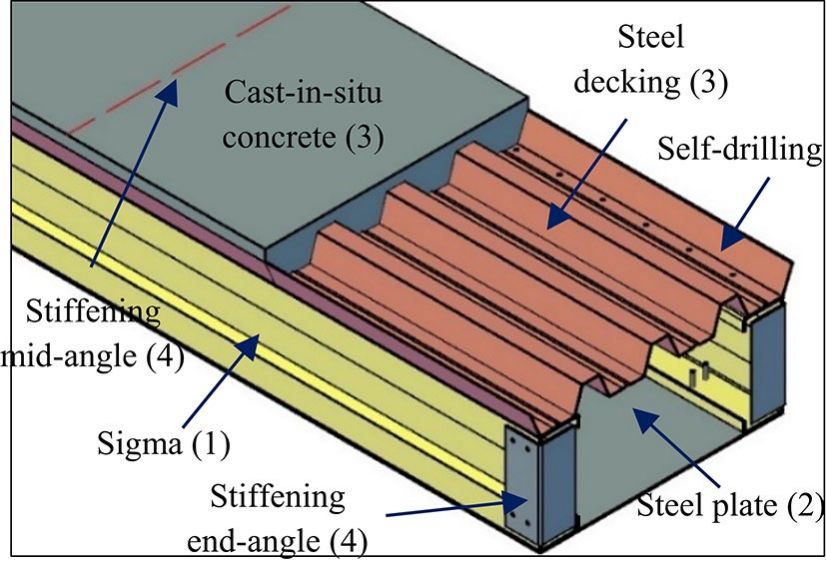
Flooring system details.

The proposed flooring system arranged in the form of a built-up box section. It consists of cold-formed steel sections, steel decking, and cast-in-situ concrete. This system has the advantage of being easily constructed, handled and transported. Moreover, it is economical because of high strength to weight ratio. Its components are available and it doesn’t need heavy tools to be assembled and prefabricated. This flooring system consists of four elements: the web (1), the lower (2), the upper (3), and the stiffening elements (4) as shown in Fig. 3.

**Fig. 4:**
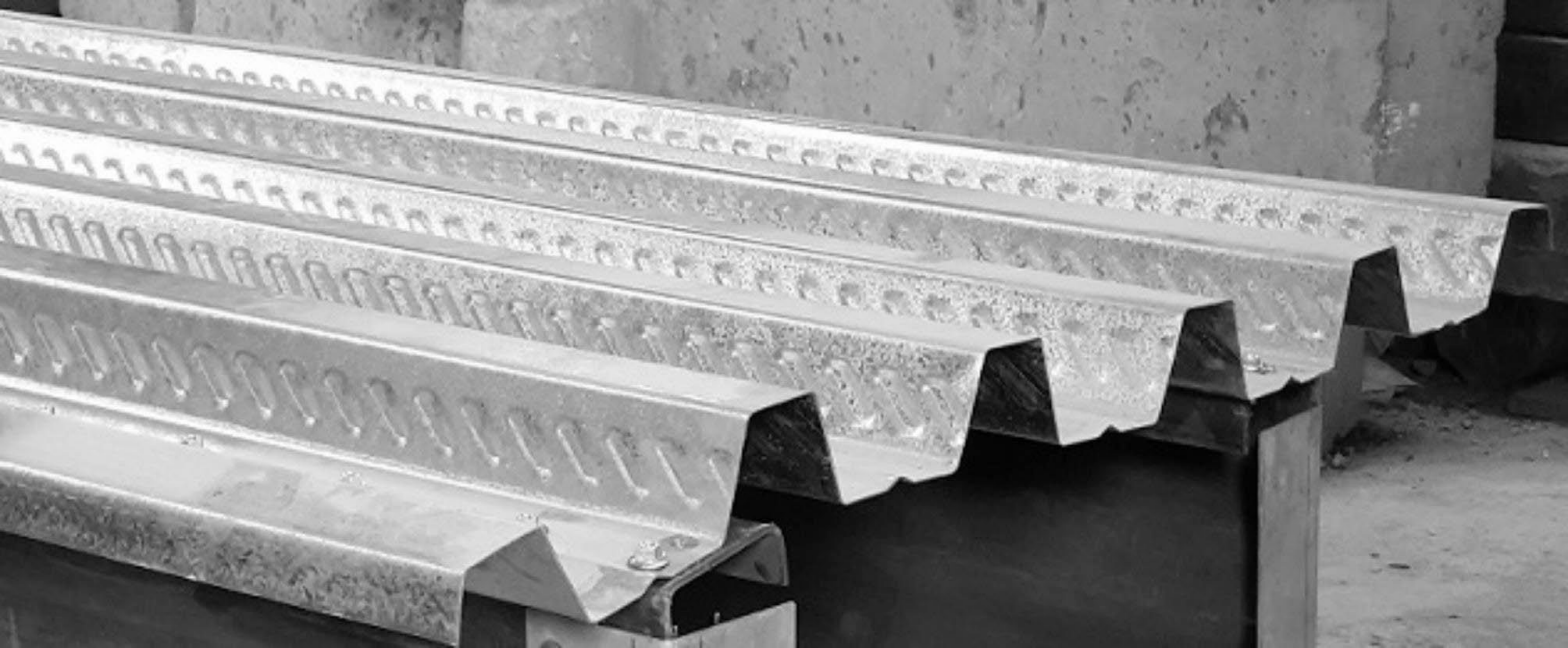
The embossments of the steel decking.

The web elements are assembled by placing two sigma sections face-to-face and arranged in a box section. The lower element is the steel plate used to fasten the lower flanges of the web elements. Moreover, it increases the tension area in the steel zone. The upper element is a composite slab composed of cast-in-situ concrete on trapezoidal steel decking. Figure 4 shows the shape of the embossments on the steel decking before pouring the concrete^32–34^. Inclined rectangle embossments increase the frictional bond strength between the steel and the concrete when dealing with longitudinal forces, making the composite deck slab a stronger system.

The system’s stiffening end and middle elements are installed. The stiffening end elements are two stiffening angles located at each support of the flooring system. Each angle is fastened by self-drilling into both ends of the sigma cross-section. The stiffening end elements increase the strength at the point of maximum shear. Additionally, one angle is placed at the mid-span of the steel decking. It used to prevent the deformation of the steel decking during concrete pouring.

The main objective of this study is to propose an alternative built-up, cold-formed flooring system that enables rapid construction while reducing costs and time. Steel decking was subsequently installed in a longitudinal direction. Time is saved by the deck’s simple alignment and adjustment for self-drilling screws to attach the deck to the Sigma sections without any overlap. Also reduces the requirement for cutting and installation, which saves manpower. The proposed system has a specimen’s length of 3000 mm and a 540 mm. In the case of installed decking in the perpendicular direction, four pieces of steel decking shall be installed next to each other to achieve the same length with overlap between the decking. This led to requiring the use of twice as many screws. This system saves approximately 20% of the cost of steel decking because there is no overlap. Additionally, it reduces the requirement for screws by 25 to 50%. In addition, SDS between upper and web elements connection are proposed as shear connectors^35^.

The failure modes, ultimate capacity, load-deflection relationships, and longitudinal strain distribution established from the tests are discussed in detail. The proposed system has more restorative advantages than the market-available solution. Its construction is quick and easy to manage. It has a high strength-to-weight ratio since it doesn’t affect the structural framework of old buildings. It could be installed at the factory and easily transferred to the site with less equipment and manpower.

## Experimental work

### Description of specimens

**Fig. 5:**
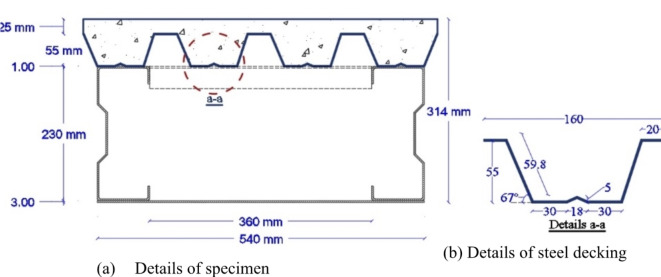
Cross-section of the specimen.

Four specimens of the proposed flooring system (CB) are studied. All specimens have the same cross-section with the following details as shown in Fig. 5a. The span (L) of the specimen is 3,000 mm and its total width (W) is 540 mm. The clear distance (W_S_) between both sigma cross sections is 360 mm. The total height (H) of the specimen is 314 mm. Furthermore, the height of the steel decking (H_d_)is 55 mm and the cast-in-situ concrete above the steel decking is 25 mm. The thickness of steel decking (t_d_) is 1 mm.The details of the one rip of the steel decking are shown in Fig. 5b. The width of the bottom steel plate is 540 mm and its thickness(t_P_)is 3 mm. The cross-section of the stiffening end angles is (80*80*3) and the stiffening mid angle is (40*40*3).

**Fig. 6:**
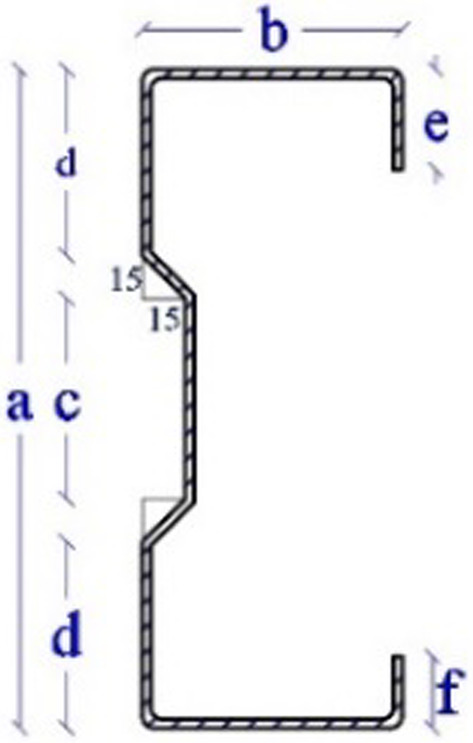
Sigma cross-section.

The specimens are divided into two groups depending on the thickness of the web element. Each group consists of two specimens. The thickness of specimens CB31 and CB32 is 3 mm, and the thickness of CB41 and CB42 is 4 mm. Details of the sigma cross sections are shown in Table 1; Fig. 6.

**Table 1:** Details of sigma cross-section.

Group	Sigma	a (mm)	c (mm)	d (mm)	b (mm)	e (mm)	f (mm)	t (mm)
(1)	CB31	230	70	65	90	30	25	3
	CB32	230	70	65	90	30	25	3
(2)	CB41	230	70	65	90	35	30	4
	CB42	230	70	65	90	35	30	4

**Fig. 7:**
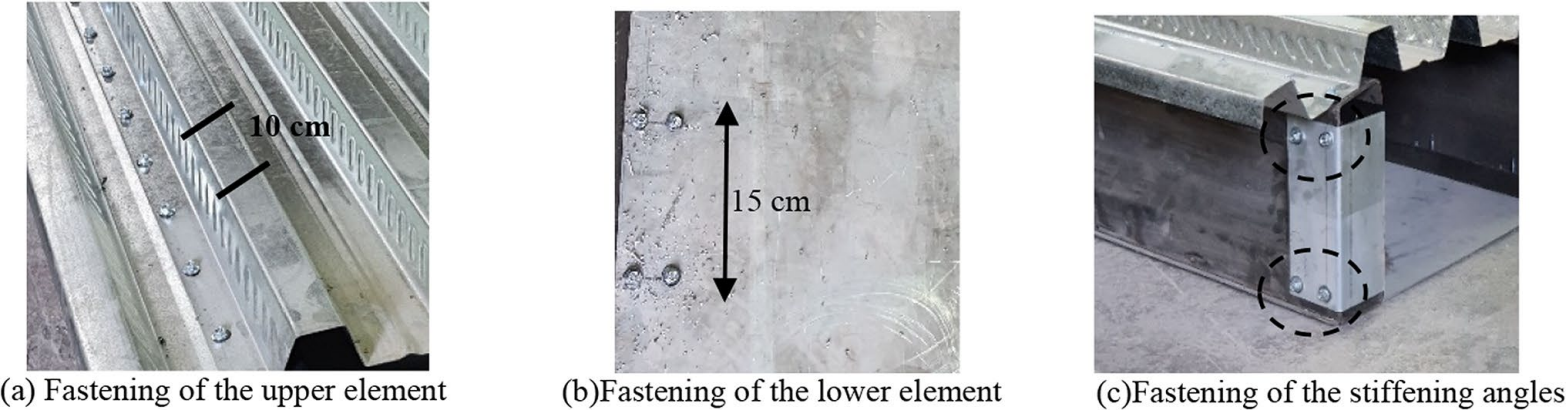
Self-drilling screws.

SDS of size (6.3 × 38 mm) are used to connect the elements of the specimens. The self-drilling screws is connected the steel decking with the upper flange of the web elements. It is arranged in one longitudinal line with a spacing of 15 cm for each side, as shown in Fig. 7a. The lower element is connected to the lower flange of the web cross section using two parallel lines of self-drillings. Additionally, it is arranged in the longitudinal direction with a spacing of 20 cm on each side, as shown in Fig. 7b. The stiffening element is connected to the end of the web element using four self-drilling screws: two screws in the upper part and two in the lower part of the end of web elements, as shown in Fig. 7c.

### Material testing

#### Tensile coupon testing

**Fig. 8:**
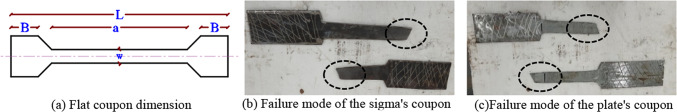
Tensile coupon testing.

**Fig. 9:**
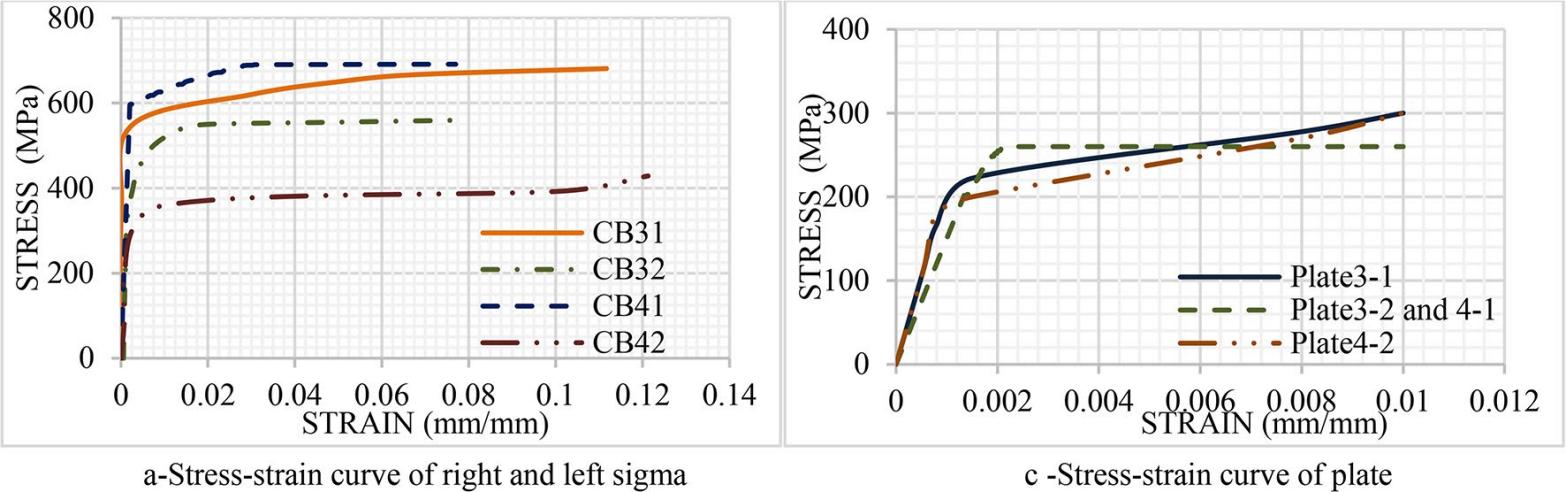
Stress-strain curve of all specimens.

All sectional dimensions were identical across four specimens. Only two different thicknesses of the sigma section were used to investigate the influence of thickness on specimen durability. As a result, the thickness of each two specimens is identical. In addition to determining the influence of material properties, the tensile coupon test was conducted accurately. In the current study, three different cross-sections were used. The material properties of sigma section and steel plate were obtained by performing tensile coupon tests. Material tensile tests are carried out for each specimen. Three tests were conducted for right and left sigma, and steel plate. This testing assessed the mechanical properties of the cold-formed steel sections. The static mechanical properties included Young’s modulus (E), yield strength (Fy), ultimate strength (FU), and strain at ultimate strength^36^. The tests are carried out according to ASTM A6. Tensile tests are performed on several flat coupons taken from each specimen. Two flat coupons from web elements (right and left sigma sections) and one from the lower element are extracted in the longitudinal direction. The width (W) is 3 mm of all coupons, and their length (a) is 30 mm as shown in Fig. 8a. Figure 8a. Figure 8b and c show the failure modes of the flat coupon taken from the sigma and the steel plate. Figure 9. shows the stress-strain curve for the web elements of the specimens. Table 2 illustrates the properties of all specimens. Where; (R) means the properties of the right sigma, (L) means the properties of the left sigma, and (P) means the properties of the plate.

**Table 2:** Mechanical properties of steel.

Specimen	Coupon	E KN/m^2^	F_y_ KN/mm^2^	F_U_ KN/mm^2^	Ɛu mm/mm
CB31	31-R	203	540	680	0.11
	31-L	203	540	680	0.11
	31-P	208	220	300	0.01
CB32	32-R	208	440	560	0.08
	32-L	208	440	560	0.08
	32-P	210	200	260	0.01
CB41	41-R	201	600	690	0.07
	41-L	201	600	690	0.07
	41-P	210	200	260	0.01
CB42	42-R	208	300	428	0.12
	42-L	208	300	428	0.12
	42-P	220	180	300	0.01

#### Performance of self-drillings screws connection

**Fig. 10:**
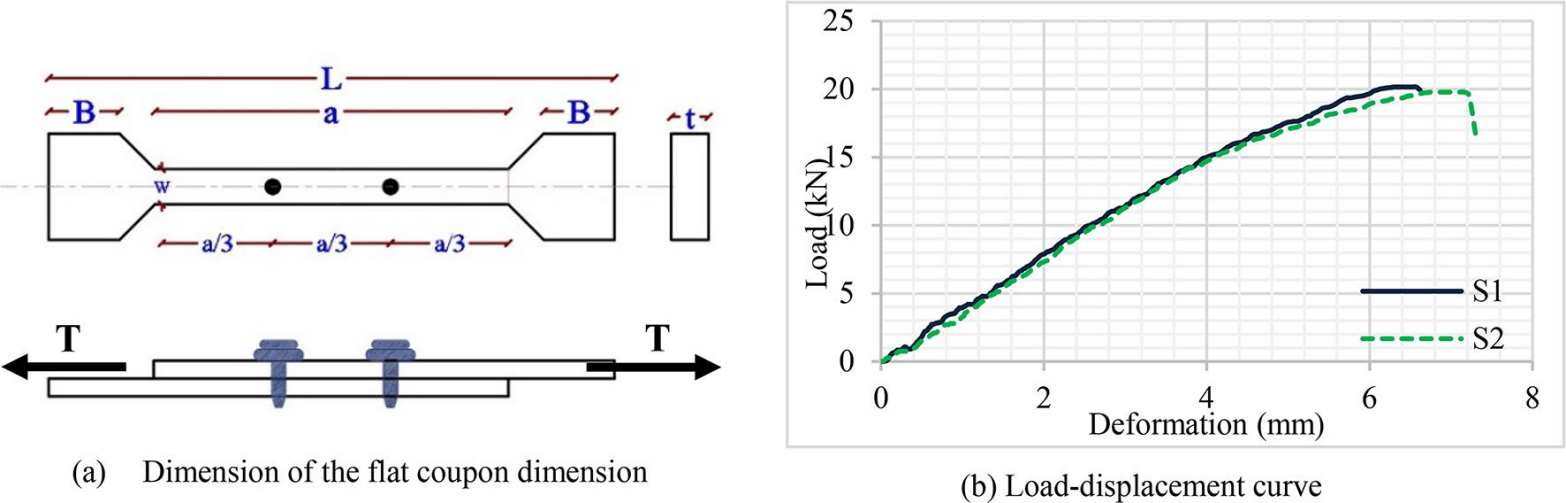
Tensile shear loading test.

**Fig. 11:**
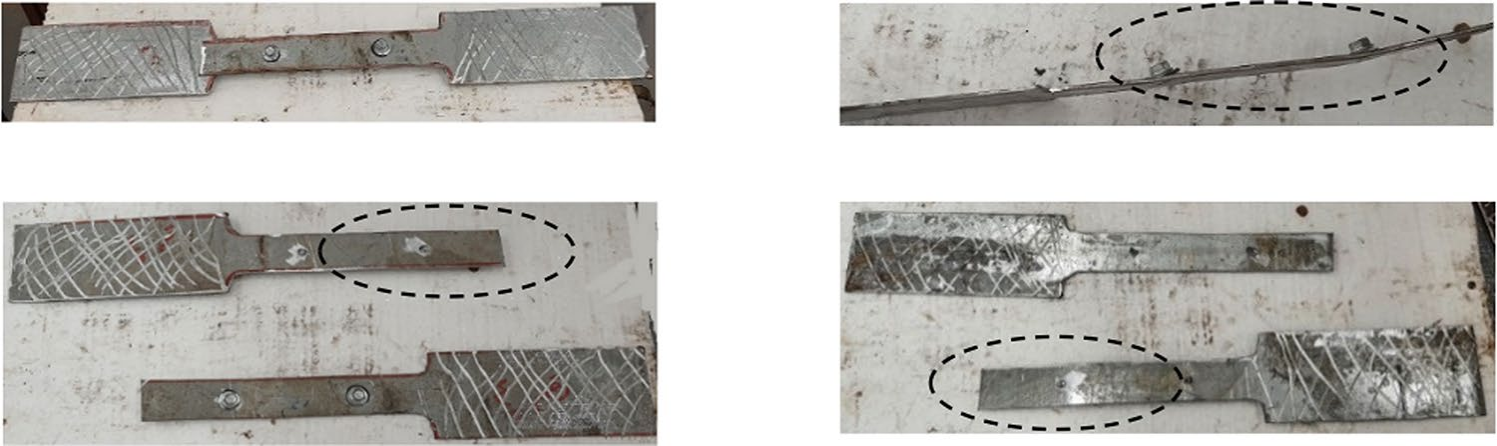
Specimens of a tensile shear loading test.

The nonlinear behavior of the force versus deformation of the SDS connections plays an important role in determining the overall structural performance. Separate single-lap shear tests are conducted to examine the mechanical performance of SDS connections. Testing coupons may fail by bearing, tilting, shearing of screws, or net-section fracture. The failure mode and strength obtained are utilized to evaluate the stiffness of the SDS connections^37^. The stiffness, shear force, and deformation capacity depend on the thickness of the steel section and the screw diameter. Single-overlap screwed coupons are prepared to represent the behavior of the connection between the steel decking and the sigma-section according to the testing methods provided in AISI S905^38^. The coupon comprises two flat coupons connected by two SDS with constant spacing, as shown in Fig. 10a. The spacing of screws should not be less than three times their diameter. The edge and end distances should exceed 1.5 times the screw diameter^39^. Two screwed flat coupons are prepared, one between the steel decking and the sigma section in group 1 and the other between the steel decking and the sigma section in group 2. All tests are performed according to ASTM A6. Figure 10b shows the load-displacement curves for a single screwed flat coupon. S1 and S2 illustrate the behavior of self-drilling screws in group 1 and 2, respectively. Subsequently, the stiffness is obtained from the load-displacement curve at the peak/yield load. The failure of the single-screwed coupons occurred due to the shear failure of screws, with the ultimate load being about 20 kN, as shown in Fig. 11.

#### Concrete strength

The mix design of M35 grade of concrete is used as illustrated in ACI324.14R-17. Five cubes of concrete are tested to check their properties. After 15 days, the first cube’s compressive strength is 30.2 MPa. The other cubes are tested after the curing period (28 days) at the same time, as the experiment was conducted. The compressive strength of CB31, CB32, CB41 and CB42 are 39.4, 38.5, 47.5 and 36.5 MPa, respectively. Table 3 illustrated all details and mechanical properties of four specimens.

**Table 3:** Details of four specimens.

	L mm	W mm	Ws mm	H mm	t mm	T_*P*_ mm	T_d_ mm	angle	E KN/m^2^	F_y_ KN/mm^2^	F_U_ KN/mm^2^	Fcu MPa
CB31	3000	540	360	230	3	3	1	80*80*3	203	540	680	39.4
CB32	3000	540	360	230	3	3	1	80*80*3	208	440	560	38.5
CB41	3000	540	360	230	4	3	1	80*80*3	201	600	690	47.5
CB42	3000	540	360	230	4	3	1	80*80*3	208	300	428	36.5

### Test setup

**Fig. 12:**
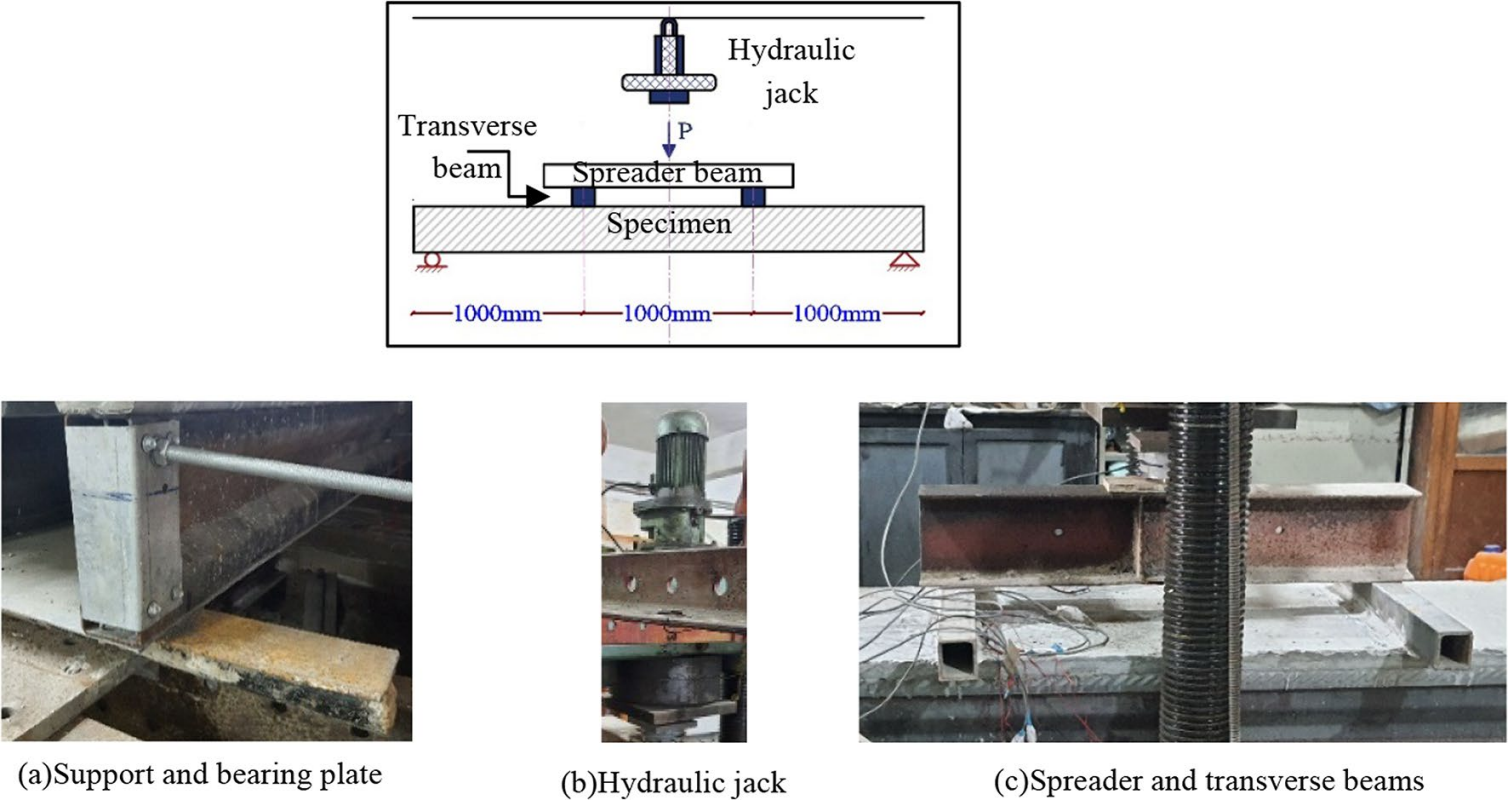
Test setup.

The experiments are conducted using a 500-kN hydraulic jack machine. All the specimens are simply supported subjected to a four-point loading system. Two transverse beams are placed on the specimen at one-third and two-thirds of the span to ensure a uniform contact surface between the load and the concrete slab. The spreader beam is set over the transverse beams and transfers the load through the transverse beam to the specimens, as shown in Fig. 12. A bearing plate was placed over the support. the bearing plate is necessary to distribute the reaction force over a sufficient length of beam to prevent web yielding or crippling. A load cell is placed between the jack and the spreader beam to measure the applied load. The rate at which a load is applied can affect how the behavior of a specimen under stress. slow loading allows them to deform plastically or absorb energy than quick loading (where they may undergo brittle failure or other types of stress). Also, If the load application is excessively fast, it may produce dynamic effects that do not accurately reflect real-world conditions, whereas slower rates may better simulate everyday conditions or service loads. Therefore, to record accurately all changes in specimens. At the beginning of the experiments the loading controller scheme had a rate of 5 kN/min. After the web element started to rotate at supports, the loading stopped for 4 min to observe the distortion development and record the experimental data. Then, the loading rate is reduced to 2 kN/min to record accurately the failure of specimens.

### Measuring system

**Fig. 13:**
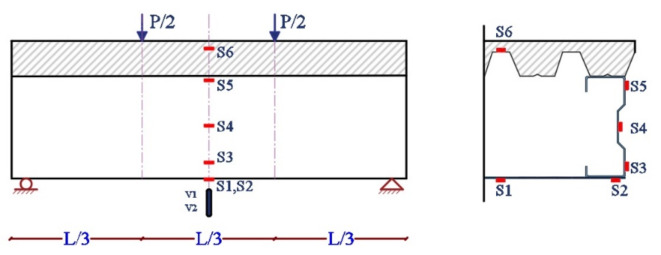
Loading and measuring system.

Four strain gauges are arranged at various locations of the midspan of each specimen. There are utilized to measure the longitudinal strain distribution along the specimen’s height at each loading stage. Two strain gauges S1 and S2 are placed at the bottom of lower element. Both of S1 and S2 at the mid span but S1 at the middle of plate and S2 at the edge. Strain gauges S3, S4, and S5 are located at the web element’s lower, middle, and upper parts. A strain gauge S6 is placed at the steel decking perforation, as shown in Fig. 11. Two linear variable differential transformers (LVDT) are used to measure the vertical deflection of the specimen at mid-span. V1 and V2 are placed in the middle of the specimen. V1 is placed on the right side and V2 on the left side (Fig. 13).

## Test results

### Failure modes

**Fig. 14:**
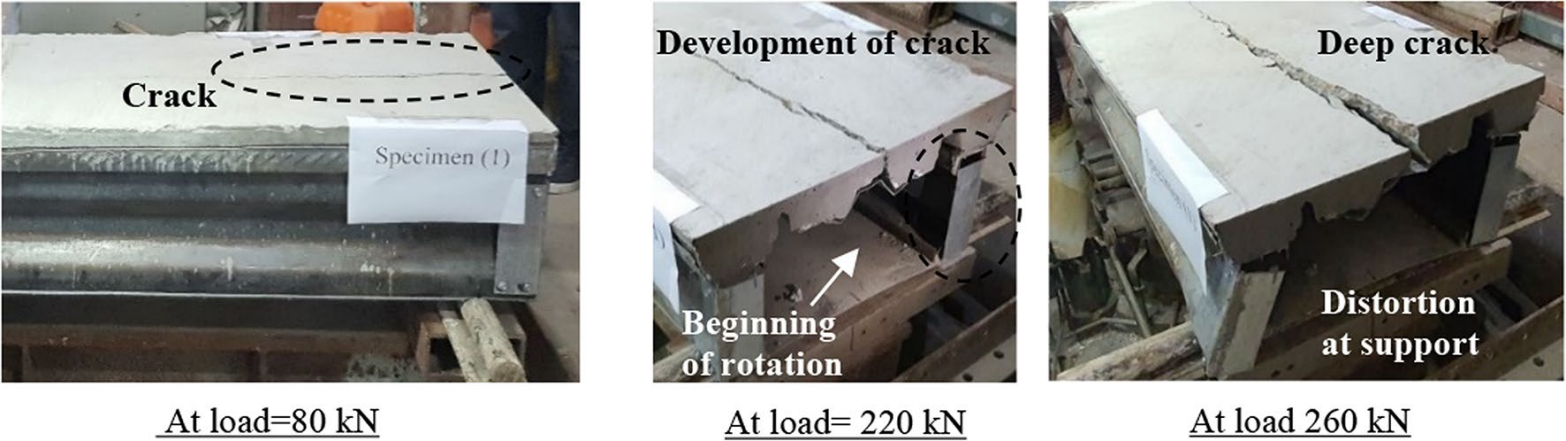
Behavior and failure mode of the specimen CB31.

The specimen (CB31) is carefully placed on the loading test machine. The strain and dial gauges are connected to the specimen. An axial compression load is applied using a hydraulic jack and gradually increased. When the applied load reached 80 kN, a fine crack appeared in the middle of the concrete at both ends. At 220 kN, the web elements of the specimen started to rotate at the both supports. Furthermore, the crack in the concrete began to expand gradually. In addition, the slip between the steel decking and the concrete started at the ends of the specimens. When the load reached 260 kN, the distortion and rotation of the web elements at supports occurred without any translation in the connection between the web elements and the upper or the lower elements. As well, the slip between the steel decking and the concrete increased, and the crack greatly expanded. Furthermore, the additional end elements also rotated with the web elements without translation. No distortions occurred in the lower element. Furthermore, no shearing failure occurred in self-drilling screws. After that, the applied load gradually decreased, and the specimen’s deformation developed more rapidly. The specimen achieved the ultimate load of 260 kN with a vertical deflection of 32 mm at mid-span (Fig. 14).

**Fig. 15:**
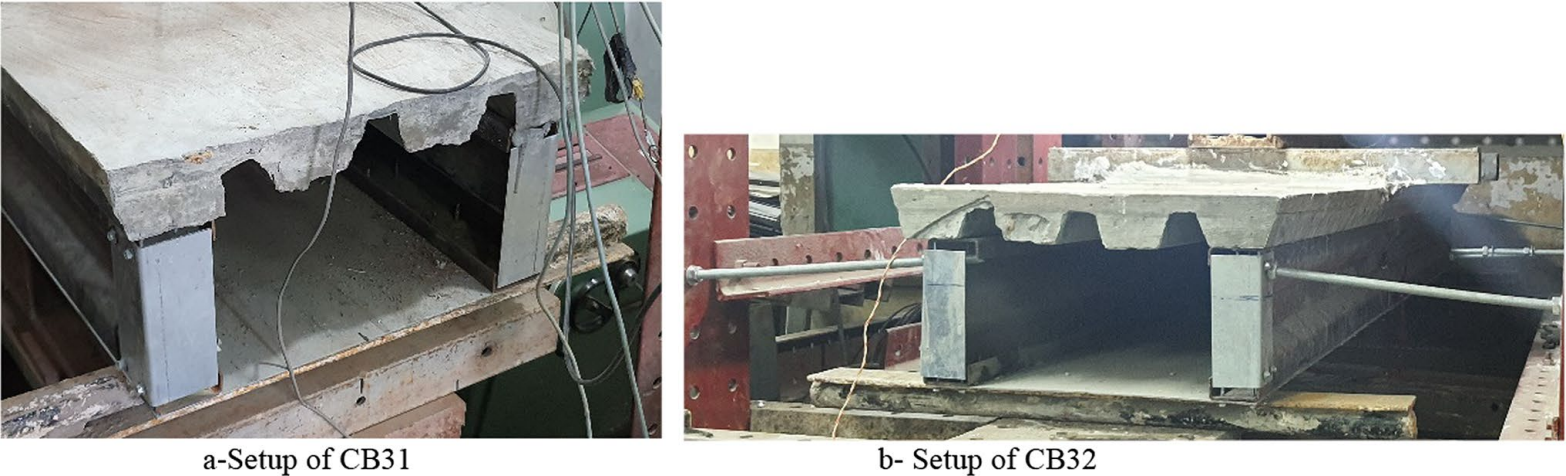
Setup of specimen.

As the first specimen CB31 is not supported laterally, the wide longitudinal crack is occurred in the middle of the concrete slab at both ends. In addition, the specimen achieved a significant distortion at the supports. Consequently, the second specimen CB32 is supported laterally by using a steel rod. The steel rod is joined between the additional end angle and the end side of the machine. The purpose of this rod is to prevent movement in the lateral direction. This simulates arranging the flooring systems next to each other in a practical site. Figure 15 illustrated the difference between the setup of Cb31 and CB32.

**Fig. 16:**
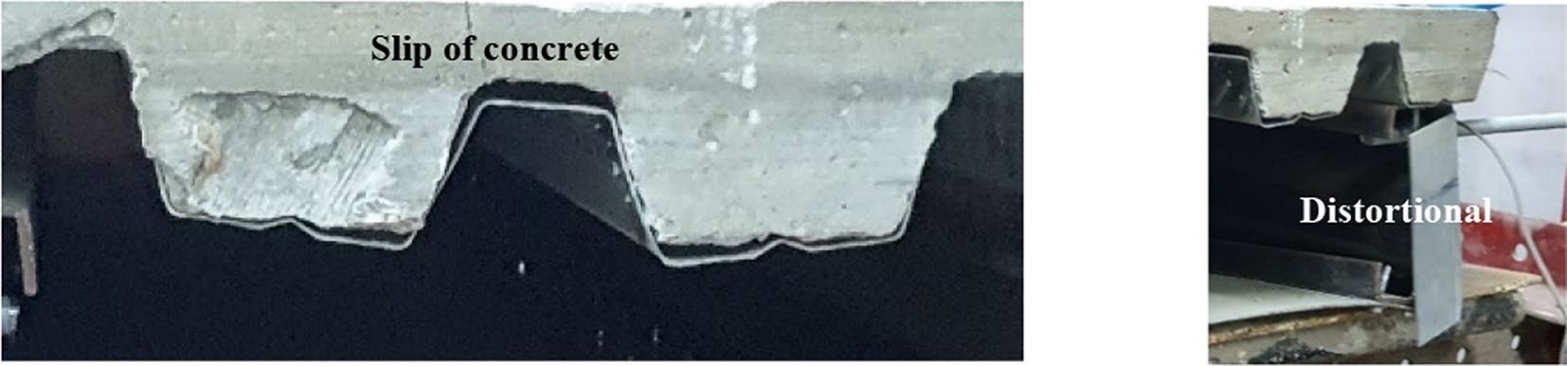
Failure mode of the specimen CB32.

The loading of CB32 had started gradually. At 130 kN, a fine longitudinal crack appeared from both ends in the middle of the concrete. At 200 kN, the rotation at the supports started, and the concrete began to slip at both ends. However, the concrete is still bonded with steel decking in the middle of the specimen. At 220 kN, the specimen’s maximum distortion is reached at both ends (Fig. 16). Then, the loading steadily decreased after that.

**Fig. 17:**
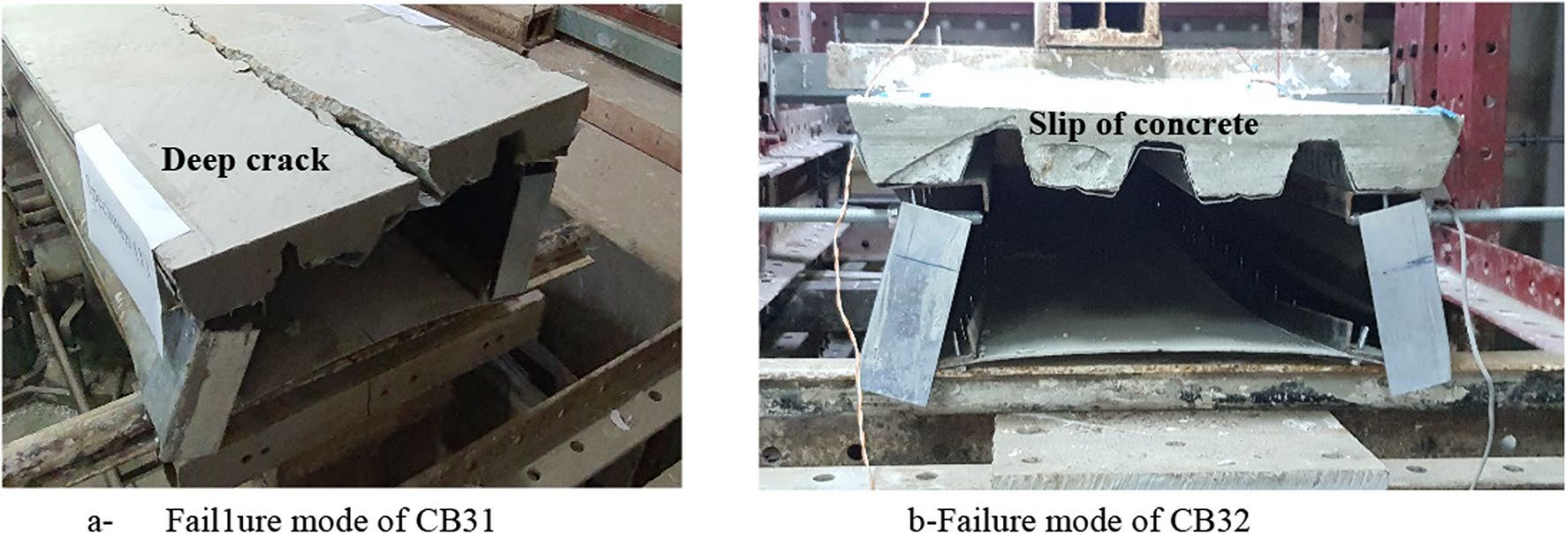
Failure mode of CB31 and CB32.

**Fig. 18:**
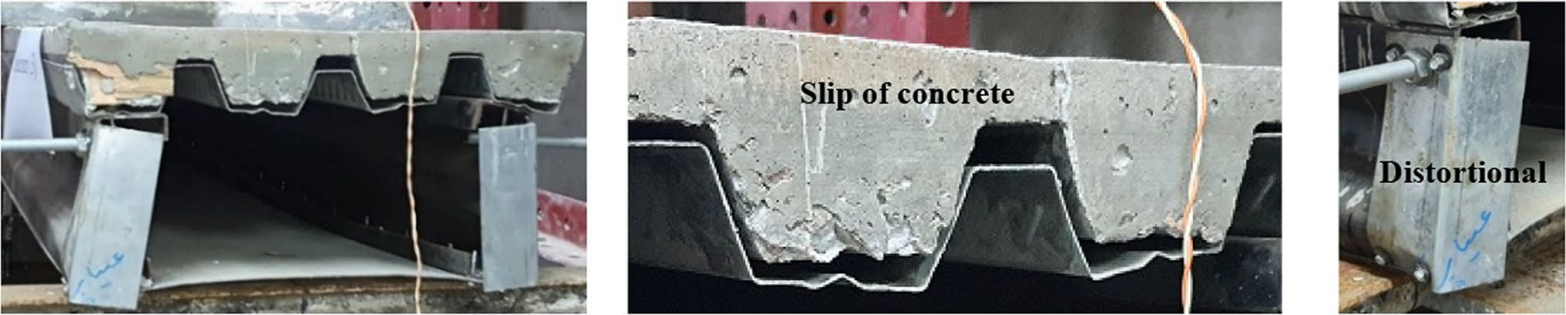
Failure mode of the specimen CB41.

**Fig. 19:**
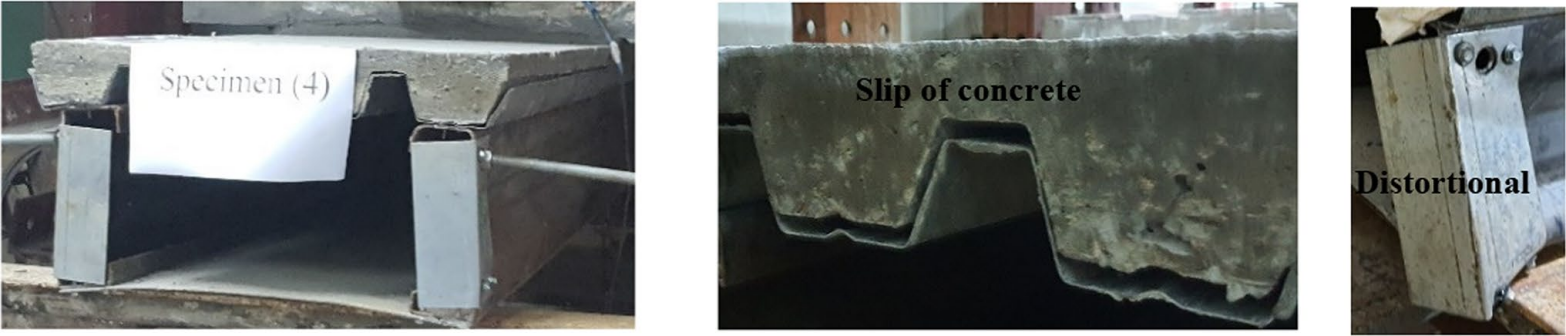
Failure mode of the specimen CB42.

Figure 17 shows the failure mode of CB31 and CB32. the difference between the two cases was that CB31, which wasn’t supported laterally, has a deep crack in concrete with maximum distortion, and CB32 has a slip on concrete with a fine crack and a little distortion at the end.

the setup of CB41 and CB42 is similar to the setup of CB32. Therefore, the experimental failure of those specimens is identical to CB32. Figures 18 and 19 show the failure mode of CB41 and CB42 respectively. the figures illustrate that the failure was due to the slip in concrete with distortion at the end without any deep crack.

In general, all specimens of flooring systems showed similar behavioral responses. The suggested systems’ failure occurred due to the slip between the steel decking and the concrete in the upper element. Then, the distortional occurred at both ends of the web element and the additional stiffening end element. There was no failure due to shear or local buckling. The lower element deformed slightly. In the first specimen group, the failure load of CB31 is 260 kN and for CB32 is 220 kN. Two factors caused the difference in failure load for both specimens. The first factor is the difference in material properties between these two specimens. Secondly, CB31 is not supported laterally, which is why CB31 encountered high distortion at both ends and a deep crack in the middle of the concrete slab. As for group 2, the failure load of CB42 is much lower than CB41 because of the difference in material properties (Table 2). Table 4 lists the specimen results, where P_1_ is the load at when the crack in the concrete started, P_2_ represents the load when the web element rotation began at both ends, i.e., the yield started, and P_U_ represents the ultimate load. M_2_ is the value of the bending moment when the rotation started, and M_U_ represents the ultimate bending moment. M_2_/M_U_ represents the yield to ultimate moment ratio. Furthermore, Table 3 records the readings of V_1_ and V_2_, which represent the maximum vertical mid-span displacements at the right and left of the specimens, respectively.

**Table 4:** Experimental results of specimens.

Group	Specimen	*P*_1_ (kN)	*P*_2_ (kN)	*P*_U_ (kN)	M_2_ (kN.m)	M_U_ (kN.m)	M_2_/M_U_	V_1_ (mm)	V_2_ (mm)
(1)	CB31	80	220	260	110	130	0.84	30.85	27.9
	CB32	130	200	220	100	110	0.90	30.2	25.6
(2)	CB41	240	320	350	160	175	0.91	34.6	32.05
	CB42	150	170	205	85	102.5	0.82	40.5	40.07

### Load-deformation curves

**Fig. 20:**
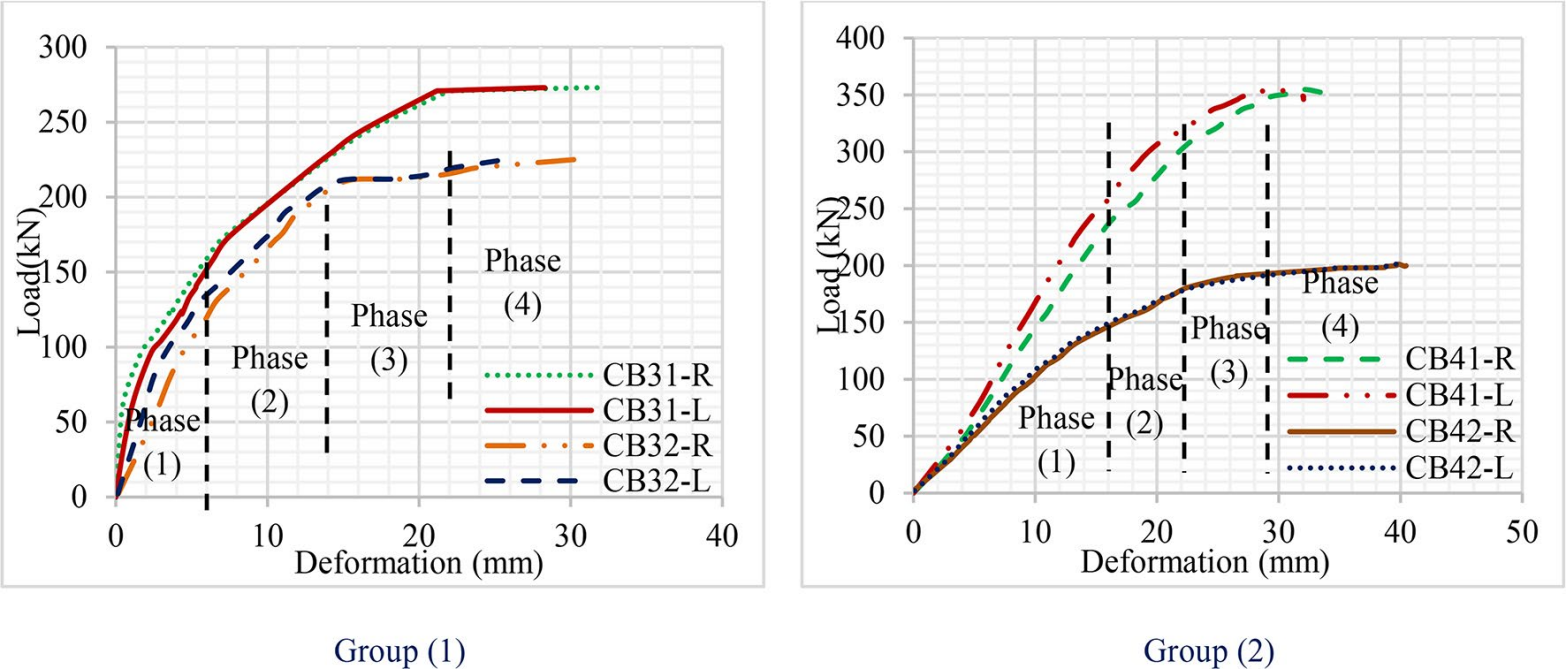
Load-deformation curve.

The load-displacement curves of all the specimens are demonstrated in Fig. 20. The curve shows the displacement recorded for both dial gauges V_1_ and V_2_. The complete loading phase is divided into four different working phases. The first one is the phase of linear elastic behavior. This phase involves the amplitude from the starting point of loading to the appearance of the first longitudinal crack in the concrete. The mid-span displacements of the four specimens develop linearly with the external force P in this phase. The second phase is the elasto-plastic phase with minor nonlinearity. The amplitude of this phase extends from the end point of phase 1 to the point at which the rotation of the web elements starts. The third phase is strong nonlinearity; the strong nonlinear part includes the amplitude from the endpoint of the weak nonlinear working (end of phase 2) to the ultimate load. The fourth phase is the failure working phase: the mid-point span displacement of specimen develops more rapidly after the third phase where the external force P declines^40^.

At group (1) : the curve demonstrates the difference in the load-deformation curves of specimens CB31 and CB32. They didn’t have a large difference in material properties, but Cb32 was supported laterally. As a result, the gap between curves started from the beginning of loading and increased gradually at the end of phase 2. At CB31, the curve increased rapidly till it reached the end of phase 3, and the curve was be constant due to the large deformation that happened with a crack in the concrete. On the opposite, CB32 increased gradually in phases 3 and 4.

At group (2): CB41 and CB42 have a large difference in material Fy of CB41 is 300 kN/mm^2^ and CB42 600 kN/mm^2^. Additionally, the two specimens are supported laterally. As a result, both curves have the same starting point but the difference appears in the middle of phase 1. The gap has widened significantly as a result of increased variations in the material’s mechanical characteristics. Because of the higher yield stress material, a huge deformation curve developed. During phase 4, CB41 and CB42 deformed to 34.6 mm and 40.5 mm, respectively, under load of 240 kN and 150 kN.

Based on all curves and data, it was established that the thickness of the sigma section has a significant impact on the durability of the specimen because specimen CB41 achieved moderate deformation with a larger load.

### Longitudinal strain distribution

**Fig. 21:**
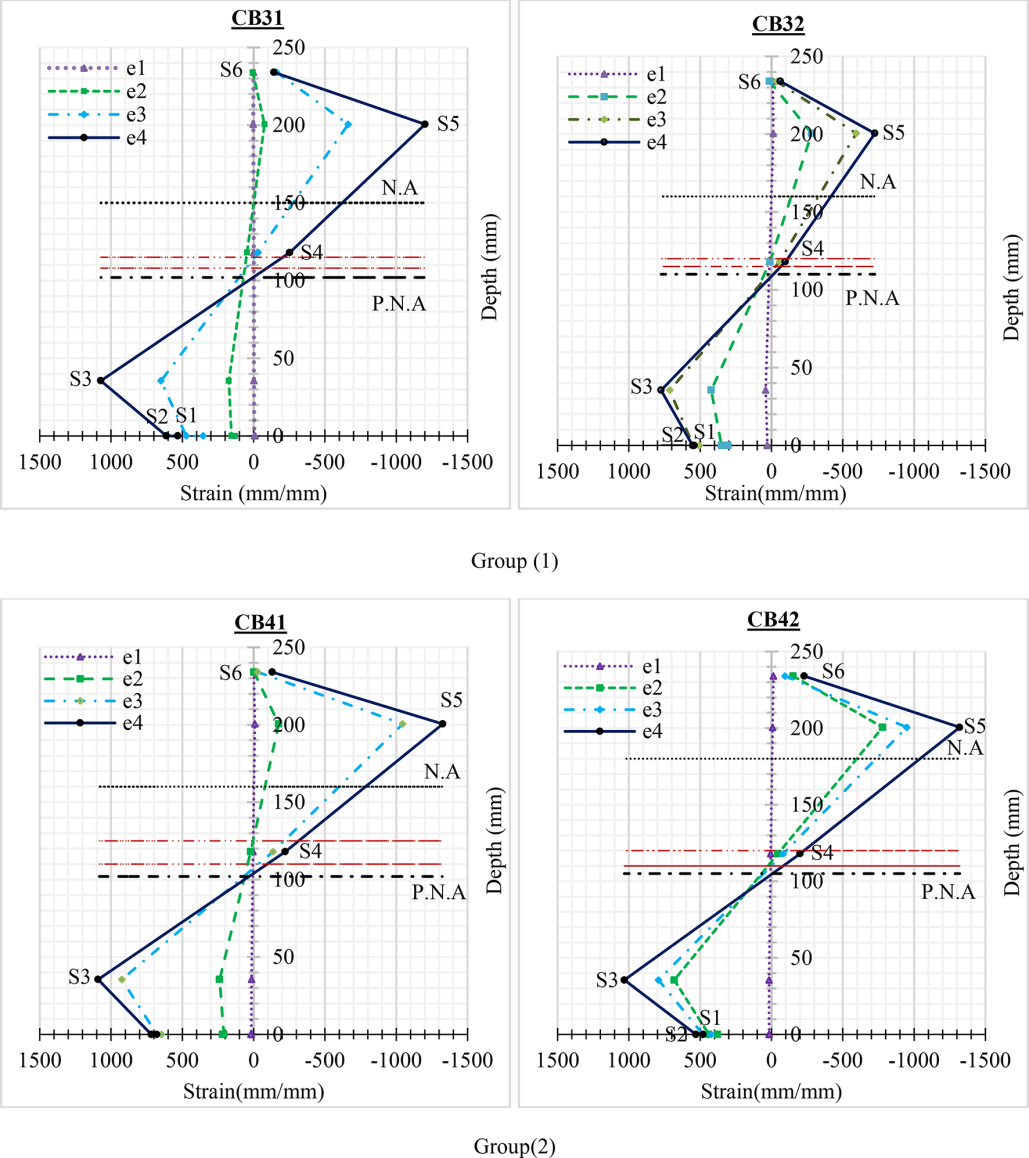
Strain distribution along the section height.

Figure 21 shows the mid-span strain distributions along the specimens’ height. Six strain gauges depicted the strain distribution at the specimens. S1 and S2 at the lower element; S3 through S5 at the web element; and S6 between steel and concert at the upper element. The strain is tracked at all times during the experiment. As a result, the strain distribution is defined in four phases: e1, e2, e3, and e4.

e1 represents phase 1 at the beginning of loading, e2 represents phase 2 when the concrete starts to crack, e3 represents phase 3 when the web elements start to rotate, and e4 represents phase 4 of the ultimate load.

The longitudinal strain (e1) is generally distributed in a linear pattern along the height of the specimen, indicating that the plane section remain plane during the test. When the concrete started to crack, the specimen’s neutral axis (N.A) moved downward (e2). In the meantime, the longitudinal strain in the upper part of the specimen increased gradually. When both ends of the web elements start to rotate, the neutral axis of the specimen also moves downwards (e3). The neutral axis moved downwards until the tension area equaled the compression area, at which the ultimate load was reached (e4).

From the curves, all specimens’ plastic neutral axis (P.N.A) is located on the web element.

The specimen CB31 not being supported laterally, the value of strain in the middle(S1) and edge (S2) of the lower element is deeply different, although being approximately comparable in other specimens. Also, the crack in the concrete that started early was led to the strain distribution e2 is nearly from e1.

The steel cold-formed section of CB32 and CB41 have higher yield strength, and also, they were supported laterally therefore the strain distribution exhibited changes across the region near the ultimate load. but CB41 has a higher strain distribution due to the difference in thickness.

The steel cold-formed section of CB42 has a lower yield strength, therefore it reaches the yield point faster. This early yielding causes a visible strain concentration about the cross-section. As a result, the specimen may show considerable permanent deformation before reaching the ultimate load.

## Determination of the ultimate flexural resistance

The bending capacity of the specimens is determined theoretically. If the results of theoretical and experimental converge, it indicates that the design represents reality well. Subsequently, the design equation is applicable to a wide range of specimens. The analytical method was calculated based on the built-up steel section only because the concrete was split at the beginning of phase 2. Then, the analytical capacity is compared with the ultimate practical results. General plastic theory can be here applied because no reduction in the case of this cross section was needed when applying the effective width concept in cold-formed.

### Ultimate flexural resistance

The ultimate flexural resistance of CB is analyzed according to the basic concepts of the plastic theory. It was used traditionally to assess the collapse behavior of structures on the basis of yielding of cross sections under proportionally increasing loading. Based on the position of the plastic neutral axis, the ultimate flexural moment capacity of the CB can be analyzed^41–43^. three assumptions are made in order to determine the maximum capacity of CB according to the AISC code^44^:

1. The tensile and compressive strengths of concrete are not considered due to the slippage between steel and concrete.
2. The compressive and tensile strength should be reduced by a reduction coefficient (λ = 0.9 for a safe design).
3. The compressive and tensile stresses of built-up steel sections could reach the yield strength.

The location of the plastic neutral axis can be determined by considering force equilibrium in the cross-section. It is defined as:

1 $$\:{A}_{st\:}{F}_{Yt}=\:{A}_{sc\:}{F}_{Yc}$$

where A_st_ and A_sc_ are the areas of the steel section in tension and compression, respectively, F_Yt_ and F_Yc_ are the yield strength of steel in tension and compression.

**Fig. 22:**
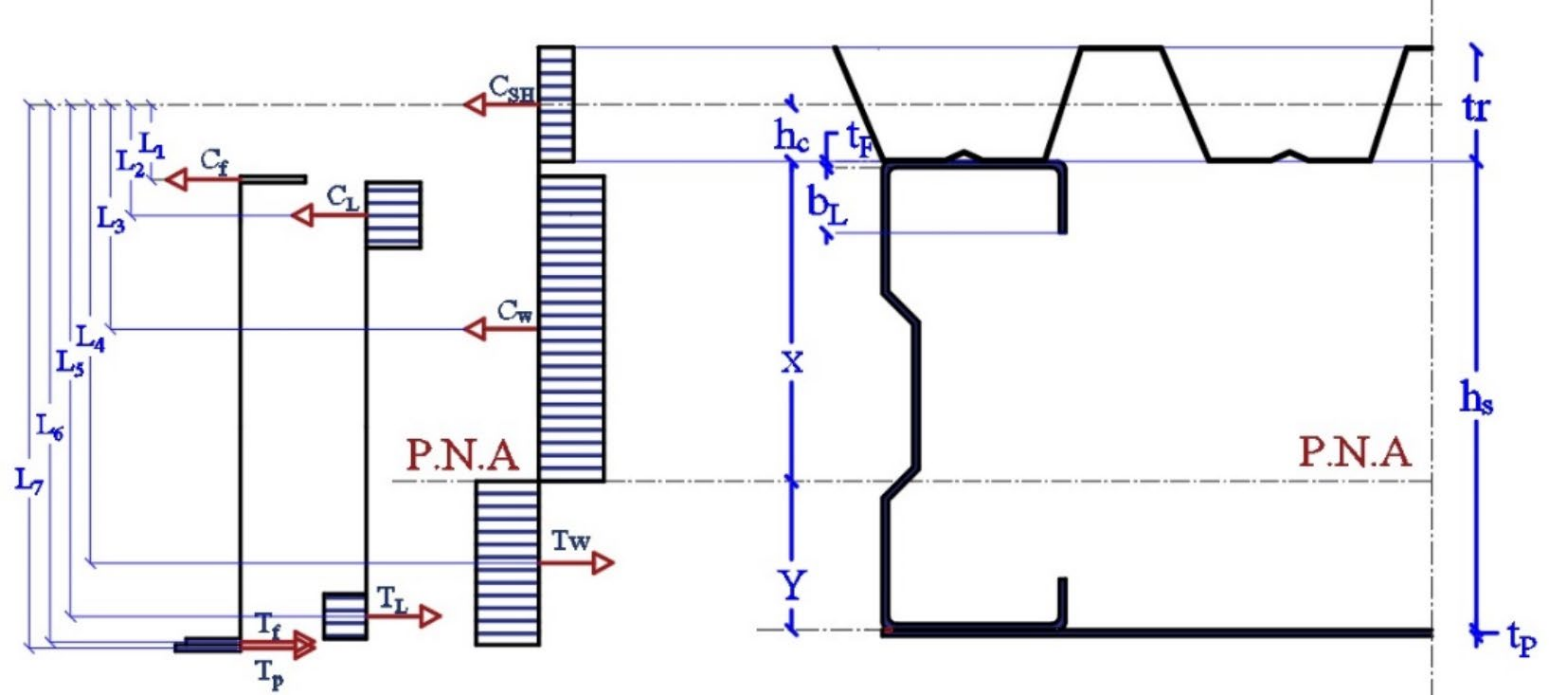
Force distribution in the built-up section.

Figure 22 shows the calculation methods and force distribution. The force is defined by area* yield strength. C_SH_ is the compression force in steel decking, C_f_ is the compression force in the sigma’s upper flange, C_L_ is the compression force in the sigma’s upper lip, C_W_ is the compression force in the sigma’s web above the neutral axis, T_W_ is the tension force in the sigma’s web below the neutral axis, T_L_ is the sigma’s tension force in the lower lip, T_f_ is the sigma’s tension force in the lower flange and T_P_ is the tension force in the lower plate.

The ultimate moment is calculated as in Eq. 2.

2 $$\:{M}_{U}=\:-\:{C}_{F}{*L}_{1}{-\:{C}_{L}{*L}_{2}-{C}_{W}{*L}_{3}+T}_{W}{*L}_{4}+{T}_{L}*\:{L}_{5}+\:{T}_{F}*\:{L}_{6}+{T}_{P}*\:{L}_{7}$$

where M_U_ = bending moment resistance, t_r_ = height of steel decking, t_f_ = thickness of the flange, h_C_ = the distance between steel decking center line to sigma section’s upper flange, b_L_= height of lip, h_S_ = height of sigma section, t_P_ = height of plate, X = position of P.N.A from the upper flange of sigma, and Y = position of (P.N.A) from te lower flange of sigma. The distance between forces illustrated in Eqs. 3–9:

3 $$\:{L}_{1}=\:{h}_{c}+{t}_{f}/2$$

4 $$\:{L}_{2}=\:{h}_{c}+{b}_{L}/2$$

5 $$\:{L}_{3}=\:{h}_{c}+X/2$$

6 $$\:{L}_{4}=\:{h}_{c}+X+Y/2$$

7 $$\:{L}_{5}=\:{h}_{c}+{h}_{s}-{b}_{L}/2$$

8 $$\:{L}_{6}=\:{h}_{c}+{h}_{s}-{t}_{f}/2\:$$

9 $$\:{L}_{7}{=\:h}_{c}+{h}_{s}+{t}_{P}/2\:$$

Table 5 compares the calculated and tested results of CB capacity to assess the accuracy of the proposed system. P_U_ and M_U_ represent the ultimate practical and moment capacity. P_US_ and M_US_ represent the built-up section’s analytical and moment capacity. Furthermore, M_U_/M_Us_ clarifies the comparison between the practical ultimate capacity and the analytical capacity of built-up section.

**Table 5:** Comparison between practical and theoretical capacity.

Specimen	Practical (Phase 3)		Analytical (Built-up section)		M_U_ /M_US_
	P_U_ kN	M_U_ kN.m	P_US_ kN	M_US_ kN.m	
CB31	260	130	242	121	1.08
CB32	220	110	198	99	1.11
CB41	350	175	327	163	1.07
CB42	205	102.5	180	90	1.14

As illustrated in Table 4, the ratio of M_U_/M_Us_ ranges from 1.05 to 1.15, with an average value of 1.07. The calculated values are in good agreement with the test results.

## Finite element analysis

In order to simulate the flexural behavior of CB, a three-dimensional finite element model is established by ANSYS^45^. The finite element method is the most powerful numerical method to study the behavior of the built-up beams^46–48^, then comparing between steel decking in parallel and transverse direction.

### Geometry and material modeling

The numerical analysis of the CB takes into account both material and geometric nonlinearities. The same mechanical properties of material of specimens were used to develop the relevant FE models. Steel is considered to be isotropic material; thus, in the ANSYS software, the mechanical properties of Young’s modulus and Poisson’s ratio were identified. Steel’s mechanical behavior, the yielding strength and the corresponding strain values were specified as illustrated before in Fig. 9; Table 2. The details of steel properties are utilized to simulate the experiment accurately. Furthermore, Concrete material is considered to be a brittle material that is usually crushed and cracked. Thus, the concrete damage plasticity option was used to identify the properties of the concrete material. the compressive strength of the material is applied as illustrated in Sect. 2.2.3^49,50^. Furthermore, the concrete component is simulated by solid element SOLID65. Cold formed steel sections are simulated by shell element ShELL181.

### Meshing and boundary condition

**Fig. 23:**
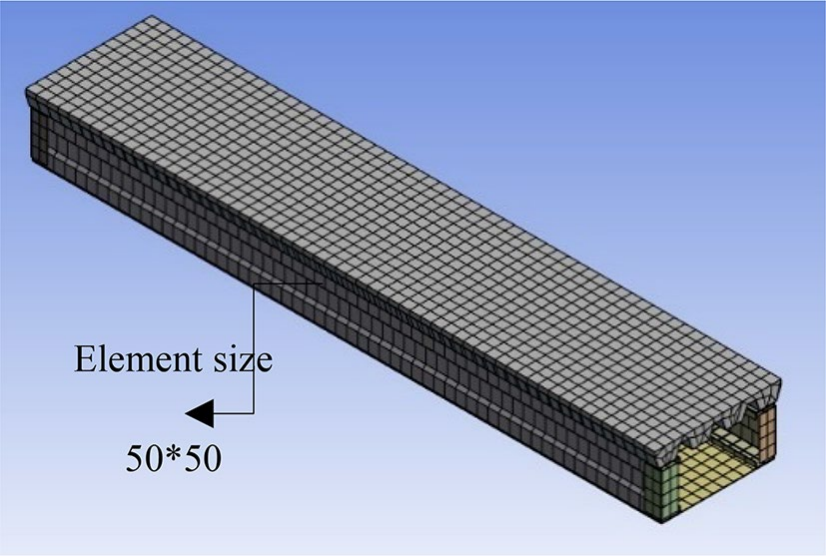
FE Meshing types.

After accomplishing more trials with various meshes, it was determined that using a constant mesh size throughout all sections of specimen provides the best validation. The linear element mesh with size (50*50) mm was used for steel and concrete. In total 6332 element were used as shown in Fig. 23. Furthermore, Table 6 illustrates the details of meshing and element type.

The modelling is simulated as a simply supported beam as a roller supported at one end and hinged at the other end. The position of the roller and hinged supports are defined at the same place in the experiments to simulate the specimen accurately. The support’s line position was allowed to rotate freely about the x-axis only to reflect the actual rolled support behavior, while the other support’s line was fully restricted against the vertical and horizontal movement to reflect the hinged support.

In addition, in specimens CB32, CB41, and CB42, we used the rod in the experiment to prevent the laterally movement, therefore it simulated as a constant movement in the z-direction.

**Fig. 24:**
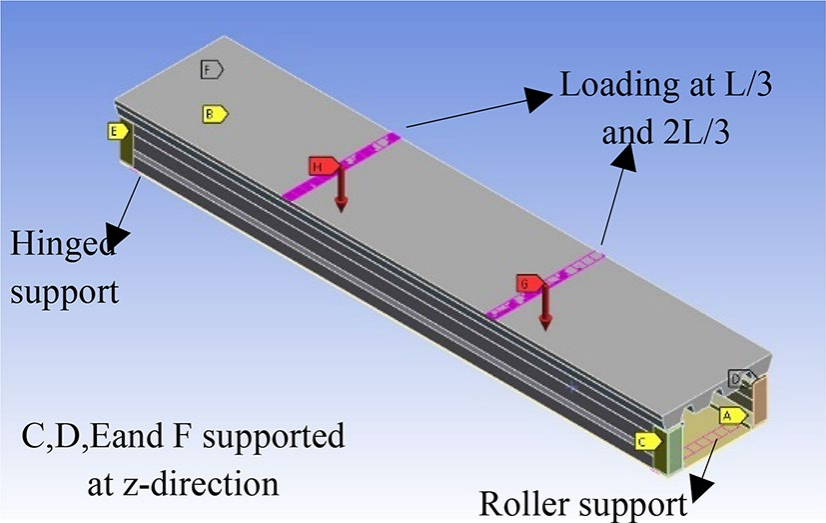
Boundary condition used in FE method.

The vertical load was applied at L/3 and 2 L/3 similar to the experiment, see Fig. 24. The actual vertical loading was implemented using an incremental downward displacement option, which was gradually increased during the FE analysis. The loading value was captured from the reaction force at the support points.

**Table 6:** Meshing and element type.

Material	Element type	Mesh Size
STEEL	SHELL 181	50*50
CONCRETE	SOLID 65	50*50

### Contact modeling

The specimen was simulated as a surface-to-surface contact option. The modelling had two types of contact: steel to steel and steel to concrete. In the steel-to-steel case, the performance of self-drilling screws, as shown in Fig. 10.b, was used. In addition, friction was used to simulate the contact between steel and concrete, similar to the experimental setting^51^.

### Initial imperfection of modeling

Initial imperfections are caused in cold-formed steel sections as a result of assembling process. The specimens undergo initial deformations due to the own weight of the metal decking and the weight of the concrete during the pouring process. For members in bending or compression two common buckling modes of importance are local buckling and distortional buckling; it is sorted into two categories: (type 1) maximum local imperfection in a stiffened element, and (type 2) maximum deviation from straightness for a lip stiffened or unstiffened flange. These imperfections are commonly considered to be in the range of L/1000 to L/2000. Due to the length of the specimen, the maximum imperfection will be evaluated and taken into consideration^52,53^.

### Verification of analysis results

**Fig. 25:**
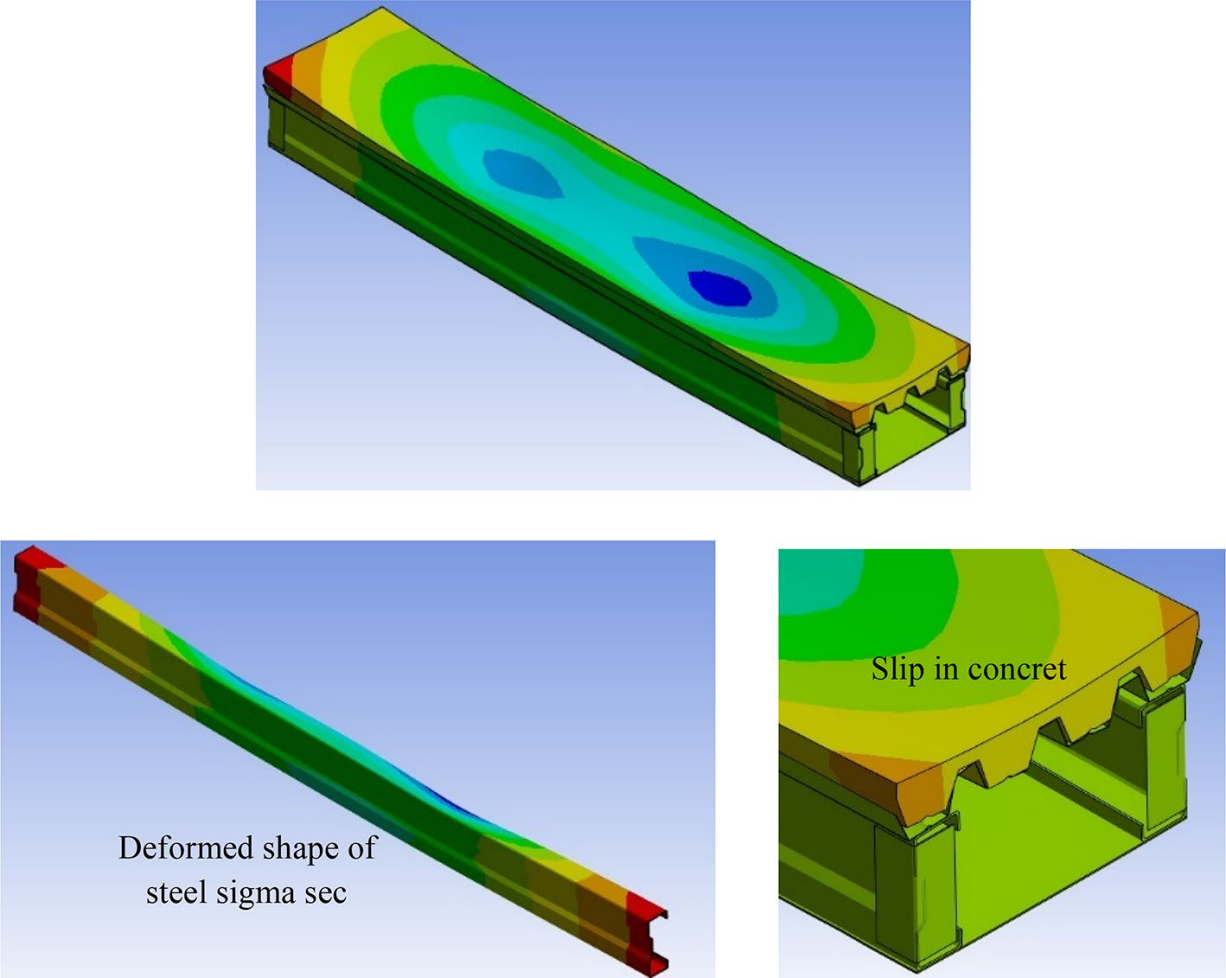
Finite element model of CB.

**Fig. 26:**
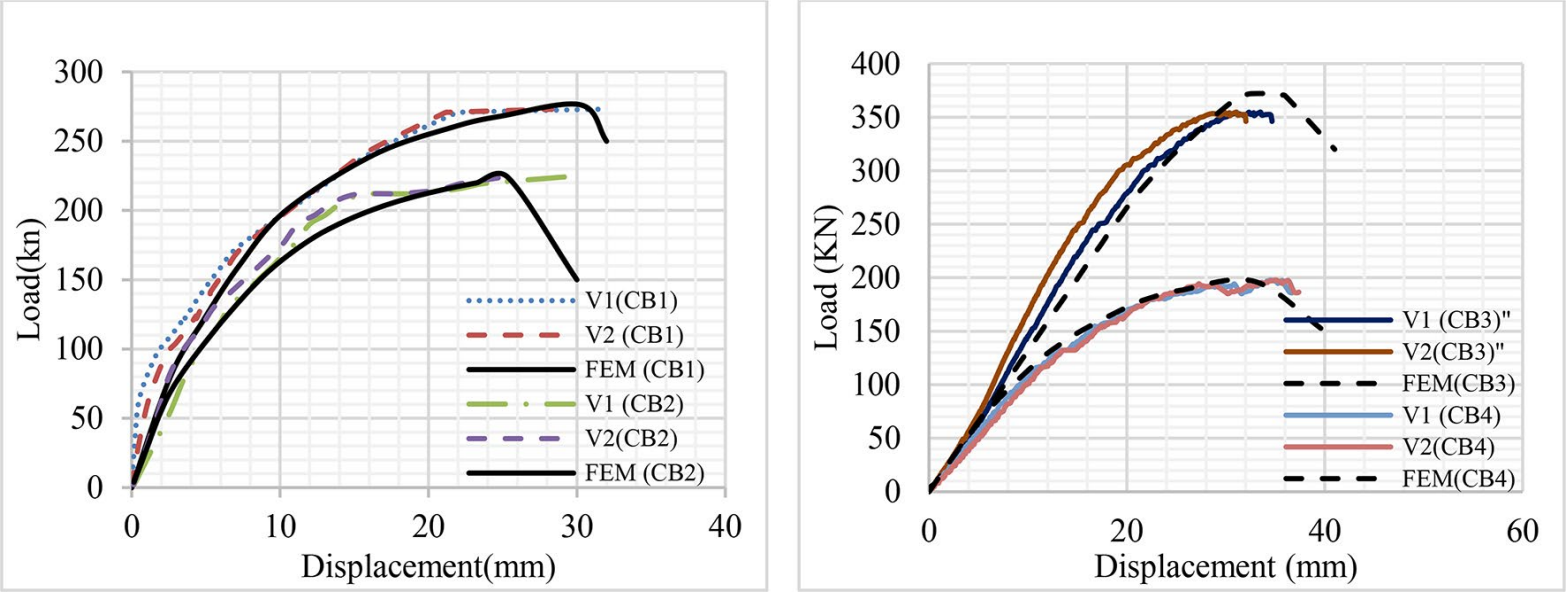
Comparison of load-displacement relation curve between Experimental and FEM.

Four simply supported CBs corresponding to the experimental are simulated by adopting the presented nonlinear numerical model for the verification objective. To further validate the FE model, the load-displacement curves and failure modes obtained from both the tests and FEA are compared in Figs. 25 and 26. The behaviour observed in the FE modelling is similar to that seen in the experiments as shown in Fig. 25. The slip between steel decking and concrete, and distortional at support without any failure due to shear or local buckling.

The load-displacement relation curves of specimens at mid-span calculated by the presented analysis model are discussed with the experimental results in Fig. 26; Table 7. Figure 26 presented the comparison between vertical displacement in right and left direction (V_1_ &V_2_) from experiments and displacement (FEM) from numerical analysis. The load-displacement curves indicated that the experiments were successfully verified using FEM. In Table 7, P_FEM_ and V_FEM_ represents the value of the ultimate load and vertical deformation from numerical analysis. Then, P_U_/P_FEM_ represent the comparison between the practical ultimate load to numerical ultimate load. The P_U_/P_FEM_ ratio were ranged from 0.94 to 1.04, with a coefficient of variation (COV) of 0.05. These statistics signify an accurate correlation between the results. Figure 26 shows that the FEA load-displacement curves are consistent with the experimental test results.

Also, these results indicated that the FEM study accurately depicts material behavior and strain distribution under loading conditions. The successful verification of the experiments with FEM explains the computational technique while also increasing confidence in the design and analysis process for similar materials and structures. Therefore, FEM can be used effectively in future simulations and assessments of specimen performance.

**Table 7:** Comparison between practical and FEM capacity.

Practical				FEM		P_U_/P_FEM_
Specimen	P_U_ (kN)	V_1_ (mm)	V_2_ (mm)	P_FEM_ (kN)	V_FEM_ (mm)	
CB31	260	30.85	27.9	276	30.25	0.94
CB32	220	30.2	25.6	223	25.36	0.98
CB41	350	34.6	32.05	372	35.02	0.94
CB42	205	40.5	40.07	196	32.94	1.04

### Comparison between the steel decking in longitudinal and transverse direction

**Fig. 27:**
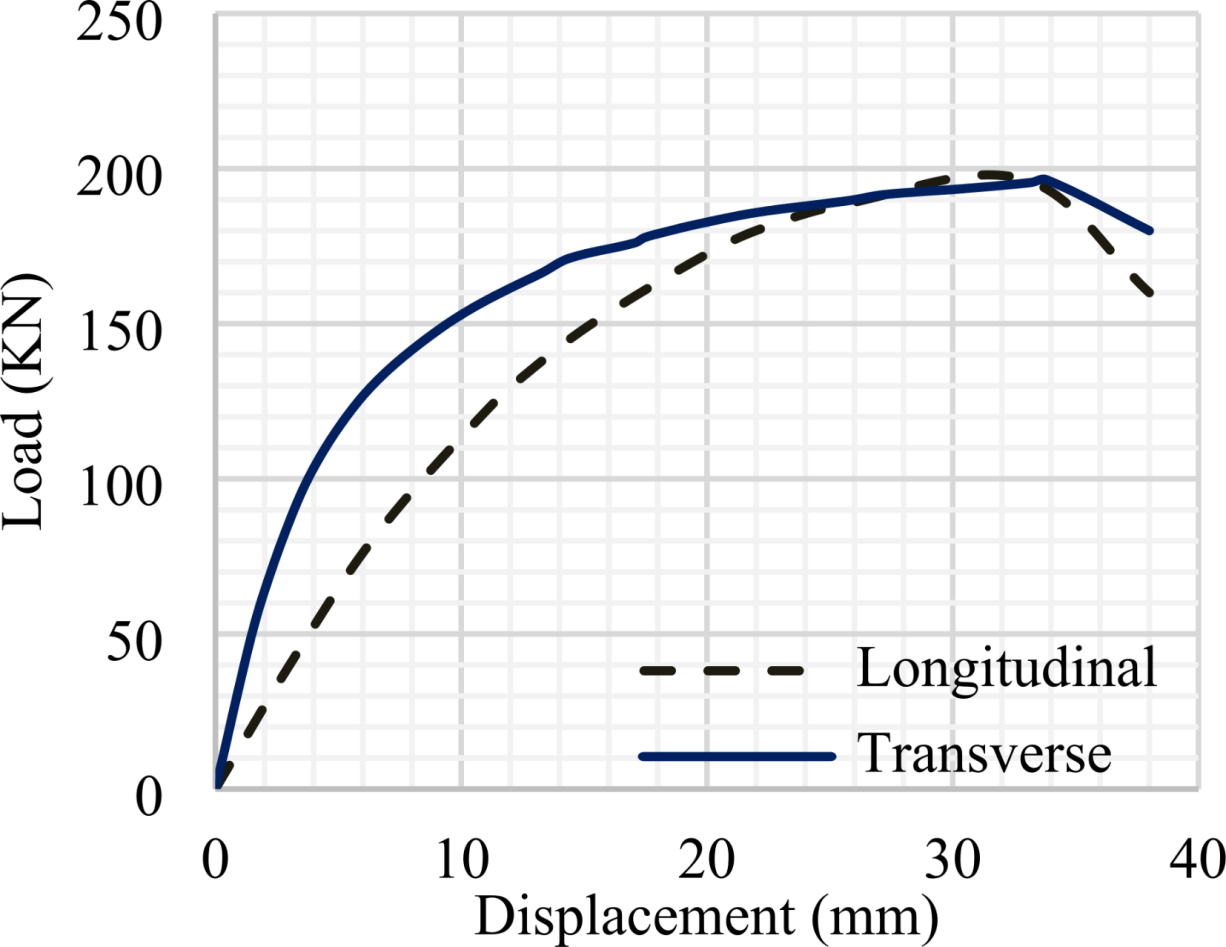
The influence of steel decking’s direction.

This study’s main objective is to suggest an alternative solution to achieve easy and fast fabrication at a low cost. Therefore, the steel decking was used in the longitudinal direction. Finite element analysis was applied to compare the steel decking in longitudinal and transverse directions Fig. 27 shows the comparison of the load-displacement curves in the longitudinal and transverse directions. The same performance of the specimen was achieved in two cases. This is due to the influence of the decking ribs’ direction when placed parallel to the specimen’s span, which can sufficiently share the bending strength with the beam part. Additionally, when the decking is in a parallel direction the composite slab, steel decking and concrete, resist the high compression stress generated at the top cross-section (mid-span) of these specimens due to the bending load. As a result, it is recommended to use each orientation based on the project’s needs and specifications.

## Conclusions

This work presents a complete experimental study on a proposed cold-formed steel flooring system. The major purpose of the proposed system is to reduce formwork and shoring needs, be lightweight and easy to handle, have quick assembly, have less equipment and depend on manpower, design flexibility, and finally durability and maintenance. This proposed system will be implemented in the restoration of old buildings which have a sturdy structure with ruined floor. Four tests were conducted to assess the behavior of this system. It consists of cold-formed steel sections and cast-in-situ concrete as a box section. This proposed system included sigma sections, steel plate, and additional angles, and the steel decking was used in the longitudinal direction, and the self-drilling screws were used to connect the steel sections. We can conclude the following:

- The four specimens have the same behavior of failure; slip between the concrete and steel decking with a fine crack at the middle of the concrete, and distortion occurs at the both ends of web elements.
- The first specimen was not supported laterally, so it has a wide crack in the concrete and high distortional at supports.
- The displacement can be classified into four phases. Moreover, all specimens exhibited high capacity and good performance in deformation. Then, the longitudinal strain distribution was illustrated.
- The mechanical qualities of the material affected the ultimate load and deformation values.
- The ultimate bending capacity of specimens was calculated theoretically according to the AISC code.
- The bending capacity was estimated in the case of built-up section.
- The comparison between practical and analytical methods was carried out and achieved good results.
- A three-dimensional finite element model has been established to study the performance of the flooring system using ANSYS software.
- The experimental data validate the analysis values gained from finite element simulation. The developed finite element models fulfill good results with specimens, including the deformation and failure modes. These findings are crucial for future numerical analysis of the newly proposed method.
- The comparison between steel decking in the longitudinal and the transverse directions was achieved using FEM.

It illustrates that the system has many useful properties in restoration due to its ease of handling and ability to be installed in the factory and transported to the site. It’s preferred to use in the old building with little equipment space and also the restored place was suitable with the dimension of the specimen. In future work, the parametric study would be used to try to change the configuration of the specimen to be used in different cases with different capacities.

## Future recommendation

- The weak bonds between steel and concrete can be enhanced in practical applications by using shear connectors. And, the thickness of concrete above the steel decking could be increased.
- Alternatively, the study of using longitudinal reinforcement steel in the composite slab can be used to attain full material strength and better performance.
- The experimental results have been validated using existing finite element models. Further on the basis of the current research, an accurate finite element models can be produced in the following step. A parametric study could be performed to identify a different size of specimen and configurations.
- Examining the impact of different CFS configurations or testing under varying environmental conditions.

## Data Availability

The datasets used and/or analysed during the current study available from the corresponding author on reasonable request.
